# Opioid Sparing Analgesics in Spine Surgery

**DOI:** 10.1155/2022/1026547

**Published:** 2022-07-30

**Authors:** Logan A. Reed, Mihir Patel, Kevin Luque, Steven M. Theiss

**Affiliations:** Department of Orthopaedic Surgery, University of Alabama at Birmingham, Birmingham, AL, USA

## Abstract

Combinations of various nonopioid analgesics have been used to decrease pain and opioid consumption postoperatively allowing for faster recovery, improved patient satisfaction, and decreased morbidity. These opioid alternatives include acetaminophen, NSAIDs, COX-2 specific inhibitors, gabapentinoids, local anesthetics, dexamethasone, and ketamine. Each of these drugs presents its own advantages and disadvantages which can make it difficult to implement universally. In addition, ambiguous administration guidelines for these nonopioid analgesics lead to a difficult implementation of standardization protocols in spine surgery. A focus on the efficacy of different pain modalities specifically within spine surgery was implemented to assist with this standardized protocol endeavor and to educate surgeons on limiting opioid prescribing in the postoperative period. The purpose of this review article is to investigate the various opioid sparing medications that have been used to decrease morbidity in spine surgery and better assist surgeons in managing postoperative pain. *Methods*. A narrative review of published literature was conducted using the search function in Google scholar and PubMed was used to narrow down search criteria. The keywords “analgesics,” “spine,” and “pain” were used.

## 1. Introduction

Surgical spine procedures can be one of the most painful operative treatments that a patient undergoes due to the extensive tissue manipulation, muscle retraction, and the substantial number of implants required for adequate fixation. These procedures necessitate significant analgesia to adequately control for pain. Moreover, inpatient postoperative pain control has been shown to be suboptimal in 57% of patients following surgical spine procedures which highlight the essential need for a more thorough evaluation of analgesic therapies in all stages of spine procedures [1]. Furthermore, over the past few decades, costs related to spine surgery have increased by 177% due to increased hospital associated costs and with the opioid epidemic that has plagued the United States, new forms and administration of nonopioid pain control are essential [2]. Since this time, the use of alternative pain sparing medications has grown exponentially in order to help offset this drastic escalation with institutions adopting various opioid sparing protocols.

Combinations of nonopioid analgesics have been proven to decrease visual analog scores (VAS) while avoiding the detrimental effects of opioid consumption postoperatively [3]. Geisler et al. presented a systematic review highlighting the importance of nonopioid analgesics and the significant reduction of opioids that results in spine surgery. However, they proposed that it was impossible to implement a gold standard for optimal treatment [4]. Opioid alternatives include acetaminophen, NSAIDs, COX-2 specific inhibitors, gabapentin, local anesthetics, dexamethasone, and ketamine. The analgesic dosage protocols vary from institution to institution; however, the general approach remains consistent with multimodal pain protocols occurring throughout the perioperative period. This review will focus on the evidence supporting nonopioid analgesia including acetaminophen, NSAIDs, COX-2 specific inhibitors, gabapentin, local anesthetics, dexamethasone, and ketamine with an emphasis on the use of these drugs in surgical spine procedures.

## 2. Acetaminophen

Acetaminophen is one of the most commonly used analgesics and antipyretic medications in the world. Despite its popularity, the mechanism of action remains unknown [5]. Historically, it has been associated with nonsteroidal anti-inflammatories (NSAIDs) due to its partial properties of inhibiting the cyclooxygenase (COX) pathways. However, it does not show significant anti-inflammatory action, interfere with platelet function, or alter renal blood flow which gives it several advantages while ultimately distinguishing it from NSAIDs [5]. The efficacy of perioperative acetaminophen in isolation has not been well studied in spine surgery. Although, IV acetaminophen has been shown to be effective in decreasing postoperative pain and overall analgesic dosage after a craniotomy [6]. Within orthopedic trauma surgery, both preoperative and intra-operative IV acetaminophen were associated with immediate postoperative pain relief and overall decreased analgesic consumption [7]. Specifically relating to spine surgery, oral acetaminophen 650 to 1000 mg can be administered preoperatively and continued postoperatively when oral intake is tolerated. The usual dose of IV acetaminophen is 650 mg every four hours or 1000 mg every six hours for patients over 50 kg with a maximum dose not to exceed 4 g/d. Although there is no set standard for these preoperative dosing regimens, one systematic review suggests a dose ranging from 300 mg to 1200 mg has shown efficacy in reducing opioid requirements and improving pain scores across a variety of spinal surgeries [8]. Interestingly, there is some evidence that when used in combination, gabapentin and acetaminophen may be more effective than when used independently. Durmus et al. found that the combination of gabapentin and acetaminophen lead to a significant decrease in pain scores when compared to gabapentin alone [9]. This further supports the evidence that combinations of these medications have synergistic effects leading to decreased patient morbidity. Taken together, there appears to be evidence towards the efficacy of a perioperative operative acetaminophen pain regimen, particularly when considering the improvement in early postoperative pain control. However, further studies focused specifically on the effect of acetaminophen along with its combination with other analgesics in patients undergoing spine surgery would be beneficial.

## 3. Alpha 2 Agonists

Clonidine and dexmedetomidine (dex) make up the alpha-2 adrenergic receptor agonists which have been widely used as a target for medical conditions such as pain disorders, hypertension, ADHD, and illicit drug use withdrawal symptoms. In recent years, alpha-2 agonists have been used as adjuncts for sedation and anesthetic use while partially blocking withdrawal symptoms in chronic opioid users [9, 10]. This advantage has made these drugs emerge as a new and effective analgesic for surgical spine patients, especially in those with opioid addiction. These medications work on a family of G-protein–coupled receptors with 3 pharmacological subtypes, alpha 2A, 2B, and 2C. Stimulation of alpha 2A and 2C subtypes in the central nervous system is believed to be responsible for the sedation and analgesic effects while the alpha 2B receptor is more commonly found in the vascular smooth muscle and is responsible for vasopressor effects [10]. This gives the added benefit of maintaining hemodynamic stability [11]. Stimulation of alpha 2 receptors in the spinal column inhibits nociceptive neurons and decreases sensitization of pain. Numerous studies using alpha-2-agonists intraoperatively have also demonstrated effectiveness in postoperative pain control following spine surgery. It has been reported that following the first 36 hours after spine surgery, morphine requirements were reduced by 43% when clonidine was administrated epidurally [12]. Furthermore, a meta-analysis of 1,200 patients revealed that intraoperative dex reduced postoperative opioid use, nausea, vomiting, as well as pain scores [13]. Ozkose et al. conducted a study of surgical spine patients with a continuous perioperative infusion of 0.2 micrograms per kg per hour which revealed a reduction in anesthetic requirements, a shorter duration of recovery, and improved pain scores [11]. Perioperative use of clonidine has also had similar results by decreasing immediate postoperative pain and opioid use, however, when compared to gabapentinoids it was less effective in this regard [14, 15]. The alpha-2 agonists also have the added benefits of decreasing intraoperative blood loss. This is seen in a study performed by Janatamakan et al. after 0.2 mg of oral clonidine was consumed right before anesthetic induction which highlighted significantly lower blood loss as compared to the control group [16]. Ultimately, these studies have provided significant evidence that alpha-2-agonist maintain significant hemodynamic advantages while decreasing pain scores and opioid consumption in the period following spine surgery.

## 4. Gabapentinoids

The gabapentinoid drug class includes gabapentin and pregabalin and has an established role in the management of neuropathic pain [17]. Gabapentin binds to the presynaptic voltage-gated calcium channels and inhibits calcium release preventing the release of excitatory neurotransmitters involved in pain [18]. They have analgesic, anticonvulsant, and anxiolytic effects which have been increasingly used as an adjunct for the management of preoperative pain. Medical uses include pain indication of diabetic neuropathy, postherpetic neuralgia, fibromyalgia, and pain associated with spinal cord injury. The general class of gabapentinoids mechanism of action is misunderstood despite being classified as a calcium channel blocker. The analgesic effects of gabapentinoids are well evident in neuropathic pain but its role in postoperative pain is less certain. Gabapentin has been shown to reduce opioid-sparing effects as well as decrease the postoperative pain scores [17]. Despite these pain sparing advantages, gabapentinoids are associated with dizziness, sedation, delirium, and respiratory depression. Because of these considerable side effects, gabapentin is not routinely used for postoperative pain control in spine surgery. Respiratory depression is a concern especially when administered with opioids for spine surgery. Despite an increased risk of overdose and respiratory complications occurring with the combination of Gabapentinoids and opioids compared to opioids alone, the absolute risk of these complications remains relatively low (e.g., for overdose, 1.4 per 10,000 patients who received both gabapentinoids and opioids, compared with 0.7 per 10,000 who received opioid alone) [19]. In addition, there is potential abuse for gabapentinoids. Despite their side effects profile, gabapentinoids are considered a well tolerable and safe drug [20]. Studies have shown a synergistic effect of gabapentin with regard to the analgesic action of morphine thus providing beneficial and clinical use in spine surgery.

A meta-analysis conducted in 2020 with 281 trials with a proportion of those trials analyzing spine procedures concluded that compared with controls, patients receiving gabapentinoids had lower absolute pain scores at 6, 12, and 24 hours following the operation [21]. However, the authors postulated that the lower scores may not correlate with clinically significant differences. The effective dose of gabapentinoids to achieve this decrease in pain and opioid consumption postoperatively may vary between surgical procedures. Although, one study did find that 600 mg within two hours prior to surgery was associated with reduced visual analog score and opioid consumption [22].

Postoperative gabapentin was associated with reduced pain scores, and a reduction in total morphine consumption and time to the first demand for analgesia was longer at 24 hours postop in patients undergoing laminectomies. Notably, this study mentioned oral gabapentin dosed at 900 mg or 1200 mg was significantly more effective than doses at 600 mg or less and placebo [23]. Interestingly, the study further went on to mention that the same efficacy of this regimen can be given during the preoperative period highlighting similar reductions in pain scores and opioid totals. Whether given preoperatively or postoperatively, gabapentin has shown a reduction in pain scores and total morphine equivalents in the first 24 hours following spine surgery and can be incorporated into any institution's pain protocol.

## 5. Glucocorticoids

Glucocorticoids are a class of steroid hormones that bind to glucosteroid receptors and induce an inhibitory effect on proinflammatory molecules and downregulate many anti-inflammatory molecules at the site of inflammation, thus leading to a suppressed immunological response [24]. Steroids are particularly useful as adjective therapy for neuropathic pain and visceral pain. Corticosteroids can directly reduce pain, reduce opioid consumption, and have beneficial symptomatic effects outside of pain relief. Glucocorticoids reduce pain by inhibiting prostaglandins synthesis, reducing inflammation, and vascular primarily leading to edema. Serious adverse effects which are often duration-related can ensue including weight gain, fat redistribution, hyperglycemia, and especially, for the purposes of our study, osteoporosis. Currently, data on the safety of dexamethasone in spine surgery is inclusive and should be individualized to specific cases even though the literature suggests benefits in specific surgical populations. Risk of perioperative corticosteroids includes hyperglycemia, impaired healing, and immunocompromised preventing its routine uses in perioperative analgesic care.

Currently, the use of exogenous dexamethasone during and after lumbar spine surgery has been controversial due to the proposed potential for increased rates of non-union. However, a meta-analysis conducted by Wang et al. included eight clinical trials with 918 patients undergoing spinal fusion and found that steroids had a positive role in reducing pain intensity and morphine consumption. Furthermore, intravenous glucocorticoid administration was associated with a reduction in length of hospital stay and the occurrence of nausea [25]. This meta-analysis also found that there was no significant difference in the risk of postoperative infection rates. Each of these studies had varying dosing regiments so the conversion of glucocorticoid equivalent to the dose of dexamethasone was required and the dose ranged from 3 to 80 mg. With the lack of an efficacious standardized dosing regimen for surgical spine procedures, further RCTs are needed to optimize dosing protocols.

## 6. Nonsteroidal Anti-inflammatory Drugs

Nonsteroidal anti-inflammatory drug (NSAID) administration has also been studied in preoperative protocols with a significant reduction in pain scores for a wide array of surgical procedures. However, controversy exists surrounding the union rates in orthopedic patients receiving nonselective Cox inhibitors, specifically Cox-2 inhibitors. Simon et al. study on animal models conclude that the Cox-2 pathway is required for adequate bone healing, discouraging NSAID use in orthopedic procedures [26]. Furthermore, in a previous study by George et al. union rates in 339,364 patients with long bone fractures were identified using private insurance claim data. Results showed that the risk of nonunion was greater in patients who had filled COX-2-inhibitor prescriptions or opioid prescriptions, but not in patients who had filled nonselective-NSAID prescriptions after the fracture [27]. Despite these findings, no consensus regarding the safety of NSAID use for orthopedic procedures exists. Future studies should aim for appropriate methodological designs to help to clarify existing discrepancies and to improve the quality of care for orthopedic patients. In the interim, cautious use of NSAIDs for fracture patients is required, keeping in mind the benefits of angelic relief and inhibition of ectopic bone formation on one hand, and the risks of nonunion on the other hand.

## 7. N-Methyl-D-Aspartate Antagonists

The N-Methyl-D-Aspartate (NMDA) antagonist ketamine has recently resurfaced in the world of anesthesia as an effective opioid complement and its use has reduced pain scores as well as opioid consumption in the postoperative period following spine procedures. A low dose postoperative infusion of ketamine is needed to bridge the transition from intraoperative tissue trauma to continued pain perception because NMDA receptors are recruited in the development and maintenance of pain sensitization after tissue traumatization [28, 29]. Notably, ketamine opposes this mechanism and works to counteract the pain sensitization that occurs following injury. Its effectiveness was highlighted in a 2011 systematic review of 70 studies that found that IV ketamine use reduced total opioid consumption and improved pain scores, particularly after major orthopedic procedures [30].

Specifically relating to spine procedures, Gorlin and colleagues determined that the most common dosing regimens included a bolus ranging from 0.15 mg/kg to 0.5 mg/kg followed by an infusion at a rate of 0.1 mg/kg to 0.2 mg/kg. They also noted that the rate of side effects tended to increase with infusions above 0.3 mg/kg [31]. Although the vast majority of research pertaining to ketamine has been done in the postoperative period, there have been several studies investigating its utility in the intraoperative periods. When considering its intra-operative use, ketamine showed short term improvements in pain scores postoperatively [32]. In a Cochrane review from 2010, Bell et al. reviewed 37 RCTs of adult surgical patients who received perioperative ketamine or placebo and found that 27 of those trials proved that ketamine reduced analgesic requirements and/or pain scores. In cervical spine surgery, intra-operative ketamine decreased opioid requirements compared to traditionally used fentanyl [33]. Importantly, among the opioid dependent population, intraoperative ketamine seems to be associated with immediate postoperative morphine consumption reduction as well as decreased use and improved pain up to 1 year postoperatively [34]. The benefits of ketamine use postoperatively have been supported in multiple studies and primarily include reduced opioid consumption immediately postoperatively and improved pain scores [35]. Similar results can be seen in the pediatric population with adolescents who received ketamine after scoliosis correction having improved pain scores, decreased opioid intake, and reduced nausea and vomiting [36].

Despite the evidence supporting the efficacy of ketamine, questions as to which dosing regimen is preferred to best reduce pain while limiting potential side effects, the timing of its administration, and the overall feasibility of its adoption into pain sparing protocols remain. Himmelseher and colleagues found that IV ketamine infused during general anesthesia may not affect pain scores if the dosing is inadequate (<0.15 mg/kg) or if the dosing schedule is insufficient [37]. In addition, this article conveys that an intraoperative bolus of 0.5 mg/kg should be followed by a low dose postoperative infusion of 0.25 mg/kg for up to 48 hours. This dosage and interval was studied in a randomized control trial which showed a significant reduction in pain scores and morphine requirements in the early postoperative recovery time period [38]. This study supports the use of a subanesthetic infusion of ketamine in the postoperative period with the authors arguing that because ketamine works by inhibiting central sensitization, the amount of ketamine should be maintained throughout the period of painful stimulation which includes during surgery and throughout the postoperative period [38].

Although a significant amount of literature supports the use of ketamine, resistance to its use has risen due to its stigma as “vet medicine,” its use as a potentially abusive drug, and the associated side effects [39]. Despite its relatively safe medication profile, side effects including sedation, dissociation, and headaches have been reported. Multiple studies investigating ketamine use in other specialties have found no significant differences in side effect profiles between patients receiving ketamine infusions and controls [40, 41]. Laskowski and colleagues found an increase in neuropsychiatric symptoms in patients receiving ketamine perioperatively, particularly at higher doses, but overall these symptoms were described to be well tolerated by most patients [30]. After adult spinal deformity correction, there was an increase in delirium among patients receiving ketamine [42]. However, ketamine has no significant effect on respiratory depression compared to traditional anesthetics [39]. Given the propensity to increase the rates of confusion and delirium, ketamine should be used with caution in the elderly, but the otherwise limited side effect profile paired with significant pain and opioid consumption reduction makes ketamine an ideal analgesic therapy for implementation in pain sparing protocols for spine procedures.

## 8. Regional Anesthesia

Regional anesthesia compromises epidural anesthesia or spinal anesthesia which provides pain sparing concentrated to the local level. Although surgical spine procedures are most often performed under general anesthesia they can be combined with these methods during the preoperative, intraoperative, and postoperative period or used alone in certain procedures to help mitigate pain. Given the extent of variability in surgical spine procedures, the administration of these techniques varies widely depending on the specific procedure being performed. Epidurals have been used in all aspects of surgical spine procedures with extensive literature reporting less opioid consumption, lower pain scores, and enhanced patient satisfaction [43]. Taenzer et al. conducted a meta-analysis of 4 studies on the use of epidurals following scoliosis surgery and found that patients without epidural analgesia had higher pain scores in the 72 hours following surgery [44]. Despite these findings supporting epidural use, critics provide evidence that side effects such as catheter loss and costs do not counteract the supposed benefit of its use for spinal surgery. Interestingly, two small studies added the benefit of an improved and more rapid return of gastrointestinal function when using epidurals for children undergoing thoracolumbar surgery [45].

Local blocks have also been documented in the literature with studies reporting significantly decreased pain scores and morphine milligram consumption postoperatively [46]. Erector spinae blocks (ESBs), although presenting a controversial mechanism of action, have been reported to provide significant benefits in spine surgery. Yayik et al. performed an RCT on thirty patients undergoing lumbar spine surgery receiving preoperative ultrasound guided 0.25% bupivacaine 20 ml ESBs and thirty patients serving as controls [46]. Furthermore, Qiu et al. conducted a systematic review of 11 studies supporting that pain scores and opioid consumption decreased postoperatively [47]. These studies ultimately concluded that ESBs reduced pain scores, opioid consumption, and extended time to first analgesics. Conversely, Tavanaei et al. reported that local intraoperative epidural use of triamcinolone acetonide-soaked Gelfoam did not provide any significant benefit in 100 patients undergoing posterolateral lumbar spinal fusion [48]. Furthermore, RCTs studies are needed which focus on novel techniques in order to implement a standardized universal protocol and mitigate the dependance on opioids.

## 9. Metamizole

Metamizole, or dipyrone, is a pyrazolone derivative acting as a muscle relaxant and analgesic with anti-inflammatory effects most commonly used in the perioperative period following any surgical procedure [49]. The antispasm muscle properties make it an ideal analgesic for application in any postoperative pain sparing spine surgery protocol. Similar to acetaminophen, the precise pharmacological pathway of Metamizole is unknown but studies have suggested that it acts by inhibiting prostaglandin synthesis in the neurological pathways [50]. Regarding the benefits of this pharmacologic in individuals undergoing spine procedures, Korkmaz Dilem et al. performed a randomized control trial and reported that pain scores reduced in the 24 hours postoperatively when compared to patients undergoing lumbar diskectomy who did not receive 1 g intravenous metamizole [51]. Interestingly, this did not have an effect on opioid consummation during this postsurgical window. Given the spasm reducing properties of the drug combined with the pain reducing benefit, metamizoles popularity has gained popularity in the European market. Metamizole in combination with the other aforementioned opioid sparing medications can help alleviate morbidity in the postoperative spine population.

## 10. Conclusion

**Figure 1:**
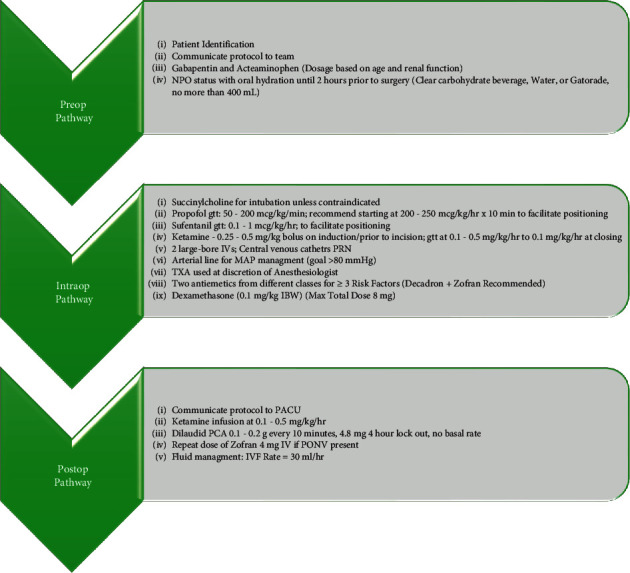
Proposed analgesic algorithm to limit opioid consumption in spine surgery.

Given the broad range of operative procedures that encompass spine surgery, it stands to reason that the more invasive procedures will require higher amounts of analgesia than others. Unfortunately, this makes a generalized standardized protocol difficult to implement. However, the current review provides a general overview of the nonopioids (Table 1) which can be used in addition to other pain sparing modalities to better assist surgeons in managing post-operative pain. We, authors, have generated a proposed protocol that would limit the use of opioids for spine surgery (Figure 1). Overall the use of acetaminophen, NSAIDs, COX-2 specific inhibitors, gabapentin, local anesthetics, dexamethasone, and ketamine have been used to mitigate narcotic consumption and improve patient satisfaction following spine surgery.

## Figures and Tables

**Table 1:** Proposed preoperative non-opioid medications and dosing regimens.

Medication	Preop dosing regimen	Benefits	Complications
Gabapentinoids	600 mg within two hours prior to surgery	Reduction of pain scores, reduction of total morphine equivalents, and longer time to first analgesics	Increased risks of sedation, respiratory depression and potentiation of the respiratory depressant effects of opioids

Acetaminophen	650 mg IV every four hours or 1000 mg every six hours IV	Increased analgesic control, decreased opioid use, and more cost-effective care	Some contraindications including severe liver disease, and some drug interactions

Glucocorticoids	IV dexamethasone 16 mg	Acute reduction of pain, improved hemodynamic stability, and decreased inflammatory response	Higher rate of wound infection

NMDA agonist	IV ketamine	Reduction of pain scores, reduction of total morphine equivalents, and longer time to first analgesics	Altered mental status
	0.15–0.5 mg/kg		

Alpha-2-agonists (dex and Clonidine)	Dex IV infusion 0.2 *µ*g/kg/hr	Decreased pain scores,	Bradycardia, hypotension, sedation
	Oral clonidine 0.2 mg	Decreased MME	Dry mouth, sedation
		Decreased EBL	

Local anesthetics	IV lidocaine	Reduction of pain and opioid usage postoperatively	CNS and CV adverse effects
	2 mg/kg/hr		

Metamizole	IV 1 g Metamizole	Anti-spasmodic, analgesic, and anti-inflammatory properties	Dyscrasias, kidney toxicity, cardiovascular toxicity, gastrointestinal toxicity, and anaphylaxis
